# Are family planning vouchers effective in increasing use, improving equity and reaching the underserved? An evaluation of a voucher program in Pakistan

**DOI:** 10.1186/s12913-019-4027-z

**Published:** 2019-03-29

**Authors:** Moazzam Ali, Syed Khurram Azmat, Hasan Bin Hamza, Md. Mizanur Rahman, Waqas Hameed

**Affiliations:** 1 Department of Reproductive Health and Research, World Health Organization, Avenue Appia 20, CH-1211 Geneva 27, Switzerland; 2 Division of Health Information Systems, Hospital for Sick Children, Toronto, Canada; 3 Department of Uro-gynecology, University of Ghent, Ghent, Belgium; 4 Health Policy, System Strengthening and Information Analysis Unit, Ministry of National Health Services, Regulations and Coordination, Islamabad, Pakistan; 5 Department of Global Health Policy, School of International Health, The University of Tokyo, Tokyo, Japan; 6 Department of Research, Monitoring and Evaluation, Marie Stopes Society, Karachi, Pakistan

**Keywords:** Vouchers, Contraceptives, Family planning, Equity, Pakistan

## Abstract

**Background:**

Low modern contraceptive prevalence rate and high unmet need in Pakistan aggravates the vulnerabilities of unintended pregnancies and births contributing to maternal morbidity and mortality. This research aims to assess the effectiveness of a free, single-purpose voucher approach in increasing the uptake, use and better targeting of modern contraceptives among women from the lowest two wealth quintiles in rural and urban communities of Punjab province, Pakistan.

**Methods:**

A quasi-interventional study with pre- and post-phases was implemented across an intervention (Chakwal) and a control district (Bhakkar) in Punjab province (August 2012–January 2015). To detect a 15% increase in modern contraceptive prevalence rate compared to baseline, 1276 women were enrolled in each arm. Difference-in-Differences (DID) estimates are reported for key variables, and concentration curves and index are described for equity.

**Results:**

Compared to baseline, awareness of contraceptives increased by 30 percentage points among population in the intervention area. Vouchers also resulted in a net increase of 16% points in current contraceptive use and 26% points in modern methods use. The underserved population demonstrated better knowledge and utilized the modern methods more than their affluent counterparts. Intervention area also reported a low method-specific discontinuation (13.7%) and high method-specific switching rates (46.6%) amongst modern contraceptive users during the past 24 months. The concentration index indicated that voucher use was more common among the poor and vouchers seem to reduce the inequality in access to modern methods across wealth quintiles.

**Conclusion:**

Vouchers can substantially expand contraceptive access and choice among the underserved populations. Vouchers are a good financing tool to improve equity, increase access, and quality of services for the underserved thus contributing towards achieving universal health coverage targets.

## Background

High population growth and fertility rates affect human development and adversely impact the health and lives of women and children [1]. Pakistan has a high total fertility rate (TFR) of 3.8 [2] and low modern contraceptive prevalence rate (mCPR) i.e. 26% [2], combined with a high unmet need of about 20%. Short-term methods are widely known and used compared to long-term methods [2].

The health system in Pakistan suffers from significant urban-rural disparities in healthcare delivery [1]. Data from the 2012–13 Pakistan Demographic Health Survey (PDHS) described that the poorest people in Pakistan, in particular rural residents, experience significant difficulty in gaining access to essential health services, including FP provided by public and private sectors [2]. The modern contraceptive uptake was 23 and 20% in the rural and in the poorest populations, respectively along with a high unmet need [1–3]. According to Pakistan Demographic Health Surveys from 1990 onwards the private sector provision of share of family planning services in the country increased from 34 to 52% specially in the rural and poorest populations [2, 4–6].

Traditionally the health sector focus has been to improve supply side with a lesser focus on utilizing demand side approaches in FP [7, 8]. Recently the emphasis has shifted towards improving the physical, financial and social access of marginalized populations to FP services using social franchising approaches including vouchers [8–19]. However, the current evidence on the effectiveness of voucher approaches seems limited [15, 17], thus highlighting the need to fill the knowledge gaps. [2, 15, 20, 21]. As most of the modern contraceptive method users obtain services through the private sector in Pakistan the lack of financial resources at the individual level can be a major impediment in acquiring FP services [2, 4].

The Family Planning 2020 (FP2020) goals are to reach 120 million more women with voluntary family planning services through the expansion of global access to family planning [22]. The intended outputs of the FP2020 goal are universal access, efficiency, quality and equity [18]. Demand Side Financing (DSF) approaches, including vouchers, aim to address some of the economic and structural barriers that limit the uptake of FP [15, 25–28] which involves transferring purchasing power to specified groups for the purchase of defined goods or services. [25, 27]. Some voucher schemes have been shown to be limitedly effective in countries like Bangladesh, India, Kenya, Indonesia, Pakistan, Ethiopia and Uganda [4, 11, 12, 15, 17, 20, 23–25, 29–31].

The paper reports a study conducted by Marie Stopes Society (MSS) Pakistan (the local affiliate of Marie Stopes International (MSI) in Pakistan) to assess the effectiveness of a free, single-purpose voucher approach (MSS model) in increasing the access, uptake, improving equity and better targeting of modern contraceptives among women from the lowest two wealth quintiles in rural and urban communities of Punjab province, Pakistan [8, 17, 32, 33].

## Methods

MSS used a combination of social franchising and voucher program to reach out to the underserved in selected areas in Punjab province, Pakistan to increase access to all methods with a special focus on long acting reversible contraceptives (LARCs). It had a quasi-interventional study design with pre and post phases implemented through an intervention, with a control arm. For the pre-intervention phase an independent cross-sectional baseline survey was conducted in May 2012 in the intervention and control arms. The intervention phase ended in January 2015, followed by an independent cross-sectional end line assessment through employing a household cross-sectional survey in the intervention and control arms between January–March 2015.

The study had an implementation arm in Chakwal and the control arm in Bhakkar districts in Punjab province, Pakistan. These districts were selected based on the basis of comparable socio-demographic and health indicators (Table 1) and distance between intervention and control districts to minimize contamination. Mianwali, was taken as second intervention district, but had to be dropped off due to security reasons and the data were omitted from the analysis (only baseline was conducted).

**Table 1:** Characteristics of intervention and control districts in Punjab province

Indicator	Selected districts in Punjab Province		Punjab Province
	Chakwal (Intervention)	Bhakkar (Control)	
Estimated population size (2011 estimated)	1,376,000	1,335,000	
% of pop. who are female aged 15–49	25	22.0	22.3
Contraceptive Prevalence Rate (CPR)	29	20	32
Modern methods	23	17.3	25.1
Traditional methods	5.5	2.7	7.1
% literate (among survey respondents)	56.7	51.3	46.6
% Wall material Kacha^a^	14	35.5	22
% Roof material Kacha	0.9	26.5	15.8
% Unemployed (among survey respondents)	12.4	6.7	6.8
Ever user of contraception but not current	6.7	3.0	4.3
Infant mortality rate	60	82	77
Under 5 mortality rate	82	119	111
% of households with television	71	49.1	63
% of households with electricity	94	90.0	92.5

^a^Kacha: not concrete/ cement

### Study intervention

Although, the details of study intervention/s package is reported in the published protocol [8]. However in brief, the MSS project is a single-purpose voucher approach and assumed that affordability was a barrier to women accessing FP services. Keeping this in mind, MSS Pakistan tested a single-purpose voucher intervention delivered to clients through an established SF network. The salient features of the intervention included the following:

- Vouchers provided only for free FP services.
- The vouchers provided three pre-paid FP visits.
- The visits were for follow-up, side-effect management and removal of FP method if required.
- Both short- and long-term contraceptive methods were provided during the visit.
- Community-level providers such as Lady Health Visitors or equivalent were trained to provide FP services, including long-term methods such as intrauterine contraceptive devices (IUDs) and implants (provided by doctors).
- Community outreach workers – called field health educators (FHE) – assessed women for poverty and the need for FP and also counselled them for FP. Women living in poverty were those who belonged to the poorest two quintiles using MSS’ poverty assessment (adapted) tool.

### Study participants

**Fig. 1:**
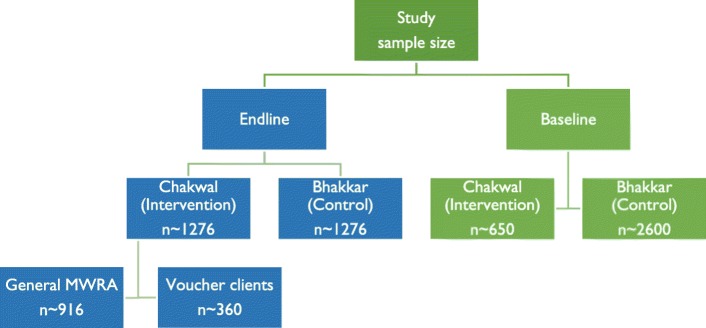
Estimated sample size distribution

Through a multi-stage sampling strategy, women aged 18–49 years (including, but not limited to post-partum women) were recruited for the baseline and end line household surveys. We also used stratified sampling in the intervention district to ensure that we recruit sufficient number of voucher clients (see Fig. 1).

### Sample size determination

The sample size was calculated assuming that the modern method CPR would increase by up to 20 percentage points from the baseline to the end line in the intervention area and that, in the control area, it would increase by 5 percentage-points between baseline and end line. Using PASS 11 software, it was estimated that group sample sizes of at least 1276 for intervention arm and 1276 for control arm would produce a two-sided 95% confidence interval for the difference in population proportions with a width of 5% when the estimated sample proportion ‘1’ is 20%, the estimated sample proportion ‘2’ is 5%, and the difference in sample proportions is 15%.

#### Stratified sampling

To ascertain success of vouchers in targeting poor women, it was estimated to have at least 360 voucher clients assuming a logistic regression of a binary response variable (Y) on a binary independent variable (X) would achieve 80% power at a 0.05 significance level to detect an odds ratio of 3.0.

We therefore used stratified sampling by recruiting respondents from the general population (strata 1) and voucher clients (strata 2). To ensure we recruited at least 360 voucher clients we allocated the required sample size (1276) for intervention district in two strata in a 3.5:1 (general population: voucher client) ratio. Therefore, we planned to recruit at least 916 respondents from the general population (strata 1) and at least 360 voucher clients (strata 2) in the intervention arm (See above Fig. 1).

### Sampling procedure

A multi-stage sampling strategy was used to recruit study participants for the end line household survey. At the first stage, catchment areas of the project clinics were refreshed from baseline within the intervention and control districts. These areas covered a 3–4 km radius around a particular clinic. The population of each catchment area was estimated at the time of the baseline survey and a list of households was developed through mapping. Each provider catchment area was defined as a cluster based on population estimates of each provider area and a list of clusters was developed. The number of Married women of reproductive age (MWRA) respondents to be selected form each cluster was determined using probability proportional to size (PPS). Finally, households from within clusters (12 clusters in each study arm) were selected from the available sampling frame of households using a simple random sampling process. One MWRA was selected for interview in each household. For voucher clients a list was prepared. The total sample size required for voucher clients was distributed across each cluster based on PPS to calculate the number of voucher clients recruited in each cluster.

### Instrument

A structured questionnaire was used that covered demographics, reproductive status, decision making and contraceptive status, quality of FP services, and poverty assessment. The questionnaire was translated from English into Urdu (national language of Pakistan) back translated, pre-tested and administered face-to-face by trained interviewers.

### Data analysis

Data were analysed using descriptive, inferential and regression statistics. Chi-square and t-test were used to compare sample characteristics between intervention and control arms to assess the differences in categorical and continuous variables respectively. Binary response variables for logistic regression analysis were contraceptive awareness (any one method), ever use (any method), current use (any method), modern method (any modern method) and first time modern contraceptive use, each recorded in Yes vs No categories. Odds ratios represented the likelihood of contraceptive - awareness, −ever use, −current use, −modern method and first time modern contraceptive use.

Effectiveness of the intervention was measured in terms of increase in modern contraceptive use and reduction in inequality by wealth quintiles in the intervention arm compared to the control from baseline to endline.

Analysis was weighted to account for the effect of oversampling of voucher clients in intervention districts. Weights were assigned based on the distribution of each stratum in the intervention area. The total estimated MWRA population in the intervention areas was 30,591, while total voucher clients (VC) in the same survey areas was 7101. The weights assigned to each stratum were 1) general population MWRA (30591–7101 = 23,490) 23490/30591 = 0.767873 and 2) voucher clients 7101/30591 = 0.232127.

To isolate the effect of the intervention, Difference-in-Differences (DID) were estimated for key contraceptive variables. Statistical Package for Social Scientists (SPSS) version 22.0 was used for data analysis.

For the equity analysis, we used household wealth index scores generated through principal component analysis. Based on baseline and endine data, quintiles were developed where quintile 1 (Q1) indicated the poorest 20% of households and quintile 5 (Q5) represented the richest. We performed the slope index of inequality [35, 36], and two relative inequality indicators (the ratio of Q5 to Q1, and the concentration index). The main interpretation of absolute index of inequalities is the difference between the extreme wealth quintiles. The relative index of inequalities is based on a ratio. The slope index (SII) uses the coverage values in the difference in percentage points between individuals at the top and bottom of the wealth scale. We calculated the SII by regressing five outcomes against an individual’s relative rank in the cumulative distribution of socioeconomic position. Concentration curves for each study area were generated to assess differences in horizontal equity over voucher client and general population. [35, 37]. Concentration index was also calculated ranging from − 1 to 1. The value of concentration index at 0, indicates that there is no inequality i.e. access to health services (utilization of modern contraceptive methods) makes no difference among poor and rich population. The negative value indicates relatively higher utilization modern contraceptive use among the poor and vice versa. All analyses at both the univariate and multiple regression stages adjusted for the probability sample weights. Equity analyses were performed using Stata version 14.0 Software (StataCorp, College Station, Texas USA).

### Ethics approval and consent to participate

All respondents were informed about the survey and their rights. No personal information was entered in the database that could be used to identify specific individuals. The study protocol was approved by National Bioethics Committee (NBC) Pakistan. Ref: No. 4–87/12/NBC-92/RDC/3548 [8]. All survey participants provided a written informed consent.

## Results

We recruited 1318 respondents from the intervention district, stratified into 390 voucher respondents and 928 respondents were from the general population. In the control district we recruited 1296 respondents from the general population.

### Demographic characteristics

#### Age structure and demographics

The average age of participating women was around 31 years in intervention and 30 years in control areas with no significant change observed in both study arms between baseline and end line including the age of husband (for details see Table 2).

**Table 2:** Demographic characteristics of participants

	Chakwal			Bhakkar		
	Baseline *n* = 691	Endline *n* = 1318	*p*-value	Baseline *n* = 2585	Endline *n* = 1296	*p*-value
	Mean ± SD			Mean ± SD		
Age of MWRA	32 ± 7.4	31.4 ± 6.3	0.057	30 ± 6.2	30.0 ± 6.6	1
Age of husband	37 ± 8.6	36.9 ± 7.3	0.784	34 ± 7.5	34.4 ± 7.8	0.123
Age of women at time of marriage	20 ± 3.7	20.4 ± 3.5	0.017	20 ± 3.3	19.6 ± 3.3	< 0.001
Male members of household (a)	3.3 ± 1.8	2.5 ± 1.2	< 0.001	3.1 ± 1.7	3.2 ± 1.7	0.085
Female members of household (b)	3.3 ± 1.8	2.4 ± 1.4	< 0.001	3.1 ± 1.8	3.1 ± 1.8	1
Average household size	6.5 ± 3.1	4.9 ± 2.1	< 0.001	6.2 ± 2.9	6.3 ± 2.8	0.308
Husband is the Head of Household	80.0	92.0	< 0.0001	87.0	75.0	< 0.001
Literacy level (%)						
Illiterate	32%	11.5%	< 0.001	63%	45.7%	< 0.001
Can read, write and perform simple sums	6%	4.2%	0.044	7%	4.4%	0.0002
Primary (1 to 5)	19%	23.9%	0.0105	15%	23.8%	< 0.0001
Middle (6 to 8)	8%	18.2%	< 0.0001	5%	9.4%	< 0.0001
Matriculation	20%	28.2%	< 0.0001	8%	9.7%	0.0368
Intermediate	6%	8.2%	0.1019	2%	2.5%	0.0517
Graduate/postgraduate	8%	5.7%	0.0883	4%	4.4%	1

Numbers are means and percentages unless otherwise specified

(a) Missing at endline Chakwal =1, Bhakkar =1, (b) baseline Chakwal =1, (c) missing endline Chakwal 16, Bhakkar 2

### Contraceptive uptake and awareness patterns

#### Current contraceptive use

Current use of any contraceptives has increased by 30 percentage points in intervention area. It increased from 21% at baseline to 51% at end line. In the control group women current use of any contraceptive at end line was 32% compared to 18% at baseline; a 14 percentage points increase at the endline (Table 3). Difference in difference analysis for contraceptive use shows the net effect for any contraceptive current user and modern method user was 16 and 26%, respectively, which was significant.

**Table 3:** Difference in difference analysis for ever use, current contraceptive use by type and method

	Control		Intervention		Absolute difference (% change)+		Net effect (% change)^
	Baseline (%)	Endline (%)	Baseline (%)	Endline (%)	Control	Intervention	
Ever user	25	58	35	79	33	44	11***
Current user ^1^	18	32	21	51	14	30	16***
Modern Method ^2^	16	22	19	50	6	32	26***
Pill	2	1	2	3	-1	1	2
IUD ^a^	2	4	2	20	2	18	16***
Injections	2	2	3	4	0	1	1
Implants		0		2	0	2	2
Condom	7	9	7	13	2	6	4*
Female sterilization	3	6	5	8	3	3	0
Traditional Method ^3^	2	10	1	1	8	0	−8****
Periodic Abstinence	2	1	0	0	−1	0	1
Withdrawal	0	3	1	0	3	−1	−4***
LAM ^b^	0	6	0	1	6	1	−5***

*P*-value: ***p < 0.01; **p < 0.05; *p < 0.1

+Absolute difference is the percentage change from baseline to endline

^Net effect is the percentage change in intervention group subtracting the percentage change in control group

^a^Intra uterine device ^b^Lactational amenorrhea method

_1_ Percentage totals % for 2 + 3

Among methods, the most significant change was observed in IUD use, which increased by 18 percentage points at end line with a net effect of 16%. The second most significant change in intervention areas was for condom use which increased by 6 percentage points.

#### Ever use and contraceptive methods awareness

Ever use of contraceptives increased significantly in project areas. In intervention areas the increase was 44% (baseline: 35%, end line: 79%) (*p* = < 0.0001) and in control areas the increase was up 33% (baseline: 25%, end line 58%) (*p* = < 0.0001), with a net change of 11% (Table 3).

At end line, awareness of methods was significantly higher in both intervention and in control areas. In the intervention areas, compared to baseline, there was a significant change in awareness of three most common methods including Pills (55 to 67%), Injections (52 to 59%) and IUDs (43 to 60%). (See in Table 4). Awareness of all contraceptive methods increased significantly in control areas.

**Table 4:** Awareness about contraceptives, overall and method specific

	Intervention			Control		
	Baseline *n* = 694	Endline *n* = 1318	*p*-value	Baseline *n* = 2582	Endline *n* = 1296	*p*-value
	%	%		%	%	
Awareness of any one method	63	93	0.0001	56	97	0.0001
Pills	55	67	< 0.0001	49	93	< 0.0001
IUD	43	60	< 0.0001	35	76	< 0.0001
Injection	52	59	0.0026	44	91	< 0.0001
Implant	18	27	< 0.0001	10	23	< 0.0001
Condom	44	65	< 0.0001	34	84	< 0.0001
Female Sterilization	36	28	0.0002	22	69	< 0.0001
Male sterilization	22	6	< 0.0001	13	18	0.0011
Emergency contraception	–	11		–	8	
Periodic abstinence	31	26	0.0561	36	16	< 0.0001
Lactational amenorrhea method (LAM)	–	78		–	64	
Withdrawal	32	14	< 0.0001	12	27	< 0.0001

Multiple responses

### Method discontinuation and switching

MWRA in intervention and control areas were inquired about contraceptive use, discontinuation and switching during the last 2 years. In Intervention district 842 women reported using a modern contraceptive method during the past 24 months. Out of these 13.7% reported discontinuing modern contraceptive use while 46.6% had switched to a modern contraceptive during the same time period. In Control district 354 women reported using a modern contraceptive method during the past 24 months. Women discontinuing modern contraceptive use in the control areas were two fold greater at 26.8% and only 13.3% had switched to using a modern method during the same time (see Table 5).

**Table 5:** Modern contraceptive use, discontinuation and switching during the last two years, measured at endline

	Intervention	Control
Respondents reporting using modern method in last 24 months	*n* = 842	*n* = 354
	n (%)	n (%)
a) Discontinuation (number of episodes)	115 (13.7)	95 (26.8)
Method discontinued	*n* = 115	*n* = 95
	*n* (%)	*n* (%)
Pills	9 (8)	9 (9)
IUD	20 (17)	17 (18)
Injection	14 (12)	13 (14)
Implant	6 (5)	–
Condom	65 (57)	55 (58)
Diaphragm	1 (1)	1 (1)
b) Switching to other modern method	392 (46.6)	47 (13.3)
Switched to different method	*n* = 392	*n* = 47
	*n* (%)	*n* (%)
Female sterilization	7 (2)	7 (15)
IUD	195 (50)	3 (6)
Injectable	37 (9)	13 (28)
Implants	21 (5)	1 (2)
Pills	22 (6)	6 (13)
Condom	108 (28)	16 (34)
Others	2	1

^a^Others = diaphragm and male sterilization

The most common methods discontinued were condoms, both in the intervention (57%) and in control (58%) areas, followed by IUD 17 and 18% in intervention and control areas respectively (see Table 5).

Women in the intervention and control areas mainly discontinued due to a desire for more children (Intervention: 81%, Control: 69%) followed by health concerns (Intervention: 13%, Control: 17%). Women who reported switching to a modern method during the last 24 months were also asked about which method they switched to. The most common methods women switched to were IUDs (50%) and condoms (28%) in intervention areas, while women in control areas most commonly switched to condoms (34%) followed by injectable contraceptives (28%).

### Targeting voucher clients

The distribution of voucher clients according to adapted poverty assessment tool indicates that 31% of the voucher clients fulfilled the poverty assessment criteria based on the tool [34]. Almost all (99,2%) of the voucher clients were FP method users. The two main FP methods used by voucher clients were IUD (56%) followed by condoms (14%).

Respondents in intervention areas were 1.8 (95% CI: 1.4–2.2), 1.7 (95% CI: 1.3–2.2) and 2.2 (95% CI: 1.7–2.8) times more likely to ever use any contraceptive method, currently use any contraceptive method and modern methods respectively as compared to respondents in the control group, adjusting for other variables in the model. Women in the ‘poorest’ socioeconomic quintile were more likely to be ever contraceptive users OR: 1.7 (95% CI: 1.2–2.4), current contraceptive users OR: 1.7 (95% CI: 1.1–2.5) and modern contraceptive users OR: 1.7 (95% CI: 1.1–2.6), compared to women in the richest quintile while adjusting for other variables in the model.,,. Women in the ‘poor’ socioeconomic quintile more likely to be ever contraceptive users OR: 1.6 (95% CI: 1.23–2.04), current contraceptive users OR: 1.4 (95% CI: 1.02–1.9), and modern contraceptive users OR: 1.4 (95% CI: 1.0–1.9), compared to women in the richest quintile while adjusting for other variables in the model (see Table 6).

**Table 6:** Logistic regression models identifying factors associated with contraceptive awareness and use among the socioeconomic quintiles (baseline and endline)

Characteristics	Contraceptive awareness any one method	Ever use (any method)	Current use (any method)	Modern method use	First time modern contraceptive use
	Odds ratio (95% Confidence interval)				
Study area					
Intervention	0.72 (0.45–1.17)	1.8 (1.44–2.24)	1.68 (1.31–2.16)	2.18 (1.67–2.84)	0.87 (0.49–1.56)
Control	1.00	1.00	1.00	1.00	1.00
Household size	1.02 (0.99–1.06)	1.07 (1.03–1.11)	1.07 (1.03–1.10)	1.07 (1.04–1.11)	0.98 (0.92–1.04)
Wealth quintile					
Poorest	1.78 (1.06–2.97)	1.68 (1.16–2.42)	1.67 (1.13–2.46)	1.69 (1.13–2.55)	0.56 (0.27–1.19)
Poor	1.26 (0.89–1.79)	1.58 (1.23–2.04)	1.37 (1.02–1.85)	1.39 (1.00–1.94)	0.62 (0.27–1.43)
Average	1.27 (0.90–1.81)	1.29 (1.03–1.62)	1.29 (0.95–1.75)	1.29 (0.93–1.80)	0.61 (0.25–1.47)
Rich	0.97 (0.76–1.24)	0.96 (0.77–1.20)	0.98 (0.76–1.27)	0.94 (0.68–1.29)	0.48 (0.21–1.08)
Richest	1.00	1.00	1.00	1.00	1.00

Adjusted for respondent’s age, husband’s age, respondent’s education, and husband’s education, household size, baseline and end line survey points

### Equity analysis

Overall mean coverage of all contraception variables including awareness, ever use, current use, modern method and first time use were substantially higher in intervention group than control group.

#### Utilization of contraceptive methods

The summary of equity indices in intervention area is shown below in Table 7.

**Table 7:** Magnitude of inequalities in contraceptive services use in the intervention areas (baseline and endline)

Characteristics		Coverage, (%)		Inequality assessment		
	Overall	Q1 (Poorest)	Q5 (Richest)	SII (Q5: Q1, % points)	RII (Q5:Q1)	Concentration index (× 100)
Intervention areas						
Ever use	58.5 (56.6–60.4)	63.4 (60.1–66.8)	27.9 (19.7–36.1)	− 23.3 (−32.4−−14.2)	0.4 (0.2–0.6)	−1.6 (− 3.2−− 0.05)
Current user	37.4 (35.4–39.4)	42.1 (38.2–45.9)	14.8 (9.6–20.0)	−18.8 (− 25.5−− 12.2)	0.5 (0.3–0.7)	−0.1 (− 2.6–2.4)
Modern method user	35.9 (33.9–37.8)	40.4 (36.7–44.2)	14.3 (9.5–19.2)	−17.5 (− 23.8−− 11.2)	0.3 (0.2–0.6)	−0.04 (− 0.8–0.7)
First time use^(a)^	68.8 (65.5–72.1)	73.7 (68.3–79.0)	78.1 (55.5–100.0)	4.4 (− 18.8–27.7)	1.2 (0.4–3.7)	− 2.3 (−5.4–0.7)
Awareness	82.6 (81.1–84.1)	88.3 (85.9–90.7)	54.2 (45.1–63.2)	− 25.5 (− 33.1−− 18.0)	0.2 (0.1–0.4)	−2.7 (− 3.8−− 1.6)
Control areas						
Ever use	36.1 (34.7–37.5)	39.3 (35.5–43.2)	35.6 (32.9–38.3)	− 12.8 (− 17.5−−8.1)	0.5 (0.4–0.7)	− 6.9 (−9.2−−4.6)
Current user	22.3 (21.0–23.6)	26.9 (23.2–30.5)	21.6 (19.2–24.0)	−9.3 (− 13.7−− 5.0)	0. 7 (0.6–0.8)	−7.0 (− 10.4−− 3.5)
Modern method user	17.7 (16.5–18.9)	22.7 (19.2–26.1)	17.2 (14.9–19.4)	−7.4 (− 11.6−− 3.2)	0.6 (0.5–0.8)	0.7 (0.3–1.1)
First time use^(a)^	76.2 (71.2–81.1)	67.6 (51.9–83.4)	82.6 (75.7–89.5)	15.0 (− 2.2–32.1)	2.3 (1.0–5.4)	5.3 (1.8–9.0)
Awareness	70.0 (68.7–71.3)	77.2 (73.9–80.6)	66.9 (64.6–69.2)	−22.1 (− 26.3−− 17.9)	0.2 (0.1–0.4)	−6.2 (− 7.3−− 5.1)

*SII* Slope index inequality, *RII* Relative index inequality; 95% CI, 95% Confidence interval, ^(a)^First time contraceptive use; All equity analysis were adjusted for baseline and end line survey point

Table 7 presents the use of contraceptive methods and maternal health services by women in the poorest and the richest quintile, based on baseline and endline data. The table also includes summary equity indices. The percentage difference (SII) of contraceptive ever use from poorest-to-richest was − 23% in intervention groups, indicating that women in the richest quintile were 23% less likely to ever use a contraceptive (Table 7).

The poorest-richest difference (SII) for current contraceptive use was − 19% in intervention areas, followed by − 18% poorest-richest difference (SII) for modern method use. Both these findings suggest that women in the richest quintiles were less likely to be current contraceptive users and modern contraceptive users respectively (Table 7).

Concentration index for awareness, modern method, and current use of contraception found negative values in intervention areas, indicating that the use of these indicators were more concentrated among the disadvantaged (poor).

#### Concentration curves and index

**Fig. 2:**
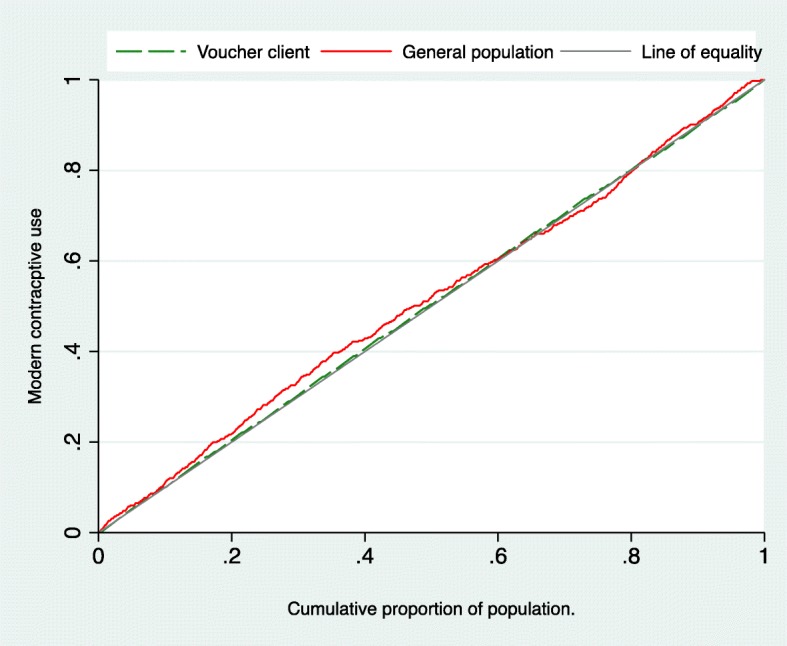
Concentration curve of modern contraceptive use by voucher client and general population

The concentration curves plot the cumulative proportion of modern contraceptives use against wealth status for intervention and control areas. The concentration curves and indices for MSS project area are presented in the below Fig. 2.

Figure Notes: Concentration index, − 0.011 (95% CI, − 0.030— 0.008; *p* = 0.24) for voucher client and − 0.030 (95% CI, − 0.071— 0.012; *p* = 0.16) for general population in the intervention arm at endline.

Concentration curves for utilization of modern contraceptive methods among voucher client and non-voucher client (general population) both lie above the line of equality, indicating a disproportionately higher concentration of modern contraceptive method use in poor population than in rich ones. However, the degree of inequality among voucher client was quite lower than general population, since the concentration curve for the voucher client lies almost closer to the equality line. For the general population, most part the concentration curves were far above the line of equality, depicting utilization of modern contraceptive methods was more pro-poor. These findings indicate that the general population in lower income have a greater proportion of modern contraceptive use than those with higher income. The concentration index for utilization of modern contraceptive use decreased from − 0.030 (95% CI, − 0.071-0.012; *p* = 0.16) in general population area to − 0.011 (95% CI, − 0.030-0.008; *p* = 0.24) in voucher client area.

## Discussion

Vouchers can be a highly effective tool to increase access to and use of family planning and reproductive health services, especially for underserved populations including the poor, youth, and postpartum women [25]. The experience in Pakistan shows that vouchers can facilitate access to modern contraceptive services where supply-side approaches don’t work. In line with other studies, the results of this study also confirmed that when vouchers are targeted towards poor beneficiaries who otherwise would not capitalise on a service - they are particularly effective at improving equity [25, 26]. Voucher programmes improve access to institutional delivery, as shown by a Cambodian voucher scheme. It has been associated with an increase of 10 percentage points in the probability of institutional delivery, and among the poorest 40% of households, the increase in the prevalence of the probability of child birth in a public health-care facility was 15.6 percentage points [38], similar results were shown in a study in Kenya [19] and Bangladesh and Pakistan [10, 28].

The World Health Organization (WHO) has suggested that, in order to overcome the lack of contraceptive services in developing regions of the world, the implementation of contracting out, social franchising and voucher schemes are of value [39–41]. The study findings also corroborate with earlier studies in Pakistan [10–12], where it was promising to note an increase in 26% net percentage points for modern contraceptives in intervention areas and an increase of 18% point in IUD use was also noted. The approach adopted in this study is perhaps resource-intensive in terms of human and financial resources, but it has been effective in increasing IUD uptake among underserved segments of the population, whereas in comparison the national figures record a very low prevalence.

Targeting has always posed challenges for any health intervention [39, 40]. The findings from our study demonstrated that as intended, the respondents in intervention areas had low education levels, and further analysis also noted that users in the lowest two quintiles were more likely to use them than their affluent counterparts [2, 39]. The findings of this study concur with the earlier findings where, compared to the control area, the discontinuation rates for modern contraceptives decreased and increase in switching pattern was noted in the intervention areas [11, 12]. However, contraceptive use in control areas also recorded an increase. This finding in itself has important implications for policy formulation. Given that secular trends in Pakistan show that contraceptive usage is increasing, the question is whether it is increasing at a sufficient magnitude and at a reasonable enough speed in order to achieve FP2020 targets for the country.

According to a national survey in Pakistan, contraceptive discontinuation rates in Pakistan stand at 37% within the first 12 months of use [2]. Furthermore, reported major reasons for discontinuation were side effects or health concerns, followed by the desire to become pregnant and method failure. In addition, out of the total method discontinuations, 80% did not switch to another method of contraception. The same national survey also recorded an overall discontinuation rate of 26% for LARC and for IUD, within 12 months of use due to any reason. Out of these, 2/3rd of the women who had their IUDs removed did not switch to another method. Findings from our study suggests that the intervention areas were successful in keeping modern-method discontinuation low at 13.7% compared to control areas where modern method discontinuation was almost twice as high, at 26.9%, during the 24 months preceding the survey. This reported low modern-method discontinuation rate is way better than the current national estimates and from the other demand-side financing studies from Pakistan using vouchers [2, 39, 42–44]. Apart from the favourable impact on the continuation of modern contraceptive use, the intervention was also instrumental in enhancing modern-method use in terms of the greater percentage of women (46.6%) compared to 13.3% of women from control areas who switched to a modern contraceptive during the same time period. This finding suggests that the intervention has been useful in promoting continuous use of modern contraceptive methods.

Equity implies that those in need have access to health services and use them. Ensuring reliable and actionable measures of health equity is especially important for managers and other implementers in global health who aim to target the poor [45–47]. Health equity measures indicated that current use and modern method use of contraception was significantly higher in poorest group in study areas than their richest counterparts, implying that vouchers increased access and use of contraceptives among the poor. The concentration curves among voucher clients curve were closer to the perfect equality line than general population curve and the concentration index decreased implying that voucher intervention has a positive impact in reducing the inequality gap between poor and rich for the use of modern contractive methods.

The key limitations of our research are mostly related to quasi experimental study design and generalizability of the findings. However, we have ensured a control and intervention area for comparison. The study captured views of women and did not include men’s perspectives. Thus the results need to be interpreted with caution. The quasi-interventional studies present some limitations such as in controlling for some confounding variables, due to non-randomization. However, such designs are recognised for use in situations where controlled trials are not feasible due to logistical, financial or other ethical reasons. Furthermore, in order to avoid any spill over effects, we chose control and intervention areas at a distance from each other. Therefore, we are confident that the increase in the outcomes of our study is attributed to the study intervention.

## Conclusion

The findings from this study suggest that the DSF approach of free voucher provision through a SF model is effective in promoting family planning, especially through long-term contraceptive methods such as IUDs. The government should consider adopting a similar approach whereby private sector service providers in the community are trained to provide family planning services through vouchers in combination with a social franchising approach depending on available resources or funding. The desirability of this approach presents some advantages.

First, the model enables widespread access to costly, long-term family planning services. The public sector can use this model to increase the reach of their services. Second, the role of community mid-level service providers in enhancing contraceptive use is highlighted as other studies concur that they can be more effective and acceptable being part of the local community. [11, 18, 24, 43, 44]. In the Pakistani context, where health-seeking needs of women is ancillary to those of male family members, the provision of quality family planning services within the community curtails the need for the women to go to distant health facilities. Lastly, engaging public sector using vouchers to reach the poor and deserving especially in rural settings with quality services will reduce the risk of women having to seek the services from unskilled providers that would increase the medical risk of morbidity and mortality.

Scaling up of voucher delivery is instrumental to FP intervention programmes that have the potential to reduce fertility levels in line with the goals set by the United Nations (UN) Sustainable Development Goals (SDGs) and Universal Health Coverage aiming to reduce global poverty [48, 49]. Findings from this study bridge the knowledge gap on effectiveness of voucher interventions that are more useful in furthering SDGs in low and middle income countries and also cater to the needs of women in family planning by subsequently decreasing the burden of morbidity and mortality resulting from unplanned and untimely pregnancies.

In future, researchers are encouraged to study the aspects of sustainability in capturing the follow-up behaviour and practices such as method continuation in the absence of voucher intervention. This information may fill in knowledge gaps regarding future scalability and sustainability of voucher programmes.

## Abbreviations

DID: Difference-in-Differences
DSF: Demand Side Financing
FP: Family Planning
IEC: Information-Education-Communication
IUD: Intrauterine Device
LARC: Long Acting Reversible Contraceptive
mCPR: Modern Contraceptive Prevalence Rate
MSI: Marie Stopes International
MSS: Marie Stopes Society
MWRA: Married Women of Reproductive Age
PPS: Probability Proportional to Size
WHO: World Health Organization
